# Effects of TLR-2/NF-κB signaling pathway on the occurrence of degenerative knee osteoarthritis: an *in vivo* and *in vitro* study

**DOI:** 10.18632/oncotarget.16199

**Published:** 2017-03-15

**Authors:** Yi-Xun Liu, Guo-Dong Wang, Xiao Wang, Yong-Le Zhang, Tian-Lun Zhang

**Affiliations:** ^1^ Department of Orthopedic, Huaihe Hospital of Henan University, Kaifeng, China; ^2^ School of Aerospace Engineering, University of Electronic Science and Technology of China, Chengdu, China

**Keywords:** degenerative osteoarthritis, toll-like receptor 2, nuclear transcription factor-kappa B, TLR-2/NF-κB signaling pathway, matrix metalloprotease-13

## Abstract

The study aims to explore the effects of TLR-2/NF-κB signaling pathway on the occurrence of degenerative knee osteoarthritis (OA). Degenerative knee OA and normal cartilage samples were collected from patients with degenerative knee OA receiving total knee arthroplasty and amputation. Expressions of TLR-2, NF-κB and MMP-13 were determined by qRT-PCR and immunochemistry. The chondrocytes were divided into control, IL-1β, IL-1β + anti-TLR-2 and IL-1β + PDTC groups. MTT assay and flow cytometry were performed to determine proliferation and apoptosis of the chondrocytes. Expressions of TLR-2, NF-κB and MMP-13 were measured by Western blotting. ELISA was conducted to detect the expressions of related inflammatory factors. The positive expressions of TLR, NF-κB and MMP13 were associated with body mass index (BMI), family history, exercise, and WOMAC scores of OA patients. Logistic regression analysis showed that OA influencing factors were TLR, NF-κB, MMP13, BMI, family history and exercise. Compared with normal chondrocytes, the expressions of TLR-2, NF-κB, MMP-13 and related inflammatory factors increased in degenerative knee OA. The chondrocytes in the IL-1β + anti-TLR-2 and IL-1β + PDTC groups showed lower apoptosis rates than those in the IL-1β group. Compared with the control group, increased expressions of TLR-2, NF-κB, phosphorylated-NF-κB (p-NF-κB), MMP-13, IL-1, IL-6 and TNF-α were found in the IL-1β group. In the IL-1β + anti-TLR-2 and IL-1β + PDTC groups, decreased expressions of NF-κB, p-NF-κB, MMP-13, IL-1, IL-6 and TNF-α were found compared with those in the IL-1β group. TLR-2/NF-κB signaling pathway contributes to the occurrence of degenerative knee OA.

## INTRODUCTION

Osteoarthritis (OA), a degenerative joint disease, is characterized by joint pain, stiffness and swelling, degeneration of articular cartilage, intra-articular inflammation with synovitis and changes in peri-articular and sub-chondral bone [1]. OA is reported to be the fourth leading cause of disability, which affects 27% of the population in industrialized countries with age above 45 years old in 2012 [2, 3]. Incidence of OA has been suggested to increase with age, rising sharply over the age of 50 years old and leveling off after 80 years old [4]. Subchondral bone sclerosis and progressive cartilage degaradation are thought as hallmarks of OA [5]. Degeneration of articular cartilage is proven to be the obvious feature of OA that eventually causes joint destruction, because of the imbalance of anabolic activities [6, 7]. Treatment for degenerative OA usually includes pharmacological and non-pharmacological modalities [8, 9]. Although degenerative OA has poor prognosis, it is significant to explore the mechanisms for the pathogenesis and treatment of degenerative OA.

Toll-like receptors (TLRs) are evolutionarily conserved molecules that promote immune responses through recognition of microbial-associated molecular patterns (MAMPs); TLR signaling is indispensable for proper activation of immune responses during infections [10]. The TLR family is best characterized as group of innate immune receptors in terms of known ligands, functional relevance and downstream signaling pathways [11]. TLR signaling has been implicated in the pathogenesis of sepsis, asthma, atherosclerosis, autoimmune disorders [12–15]. While pathogen recognition begins at the receptor level, specific transcriptional response and immunological outcome are ultimately determined by the signaling components downstream and the way they interact with each other [16]. Nuclear factor-kappa B (NF-κB) has been recognized to be the master orchestrator of TLR-induced responses; all TLR signals converge on NF-κB and activation of NF-κB is critical for TLR function [17]. NF-κB is a transcription factor regulating gene expression, which can control multiple cellular functions, such as inflammatory and stress-induced responses and survival [18]. NF-κB is a critical regulator of innate immunity/inflammation; aberrant NF-κB regulation has involved in many cancers[19–22]. Besides, TLR2 expression increases in OA chondrocytes, and synoviocytes could produce RANKL, receptor activator of NF-κB ligand, which is involved in cartilage degradation and joint destruction [23, 24]. Although TLR-2/NF-κB signaling pathway might participate in the occurrence of degenerative OA, the inner influence mechanism is still unclear. Consistently, as a pro-inflammatory cytokine, IL-1β is a primary instigator in cartilage degradation in OA [25]. Also, previous study has proven that up-regulated matrix metalloprotease-13 (MMP-13) expression is essential in the OA pathogenesis [26]. Therefore, this study aims to explore the effects of TLR-2/NF-κB signaling pathway on the occurrence of degenerative OA.

## RESULTS

### Baseline characteristics of subjects in the OA and normal groups

Baseline characteristics of subjects in the OA and normal groups were shown in Table 1, in which age, gender, smoking, drinking and tea drinking habits had no significant difference and BMI, family history and exercise had statistically significance.

**Table 1:** Comparisons of baseline characteristics of subjects in the OA and normal groups

Index	Case group (n = 231)	Normal group (n=198)	*P* value
Age			0.336
≥ 60	174	141	
< 60	57	57	
Gender			0.368
Male	79	76	
Female	152	122	
BMI (kg/m^2^)			< 0.001
< 23	111	99	
23 ~ 25	64	81	
> 25	56	18	
Smoking			0.132
Yes	116	85	
No	115	113	
Drinking			0.196
Yes	80	57	
No	151	141	
Family history			< 0.001
Yes	138	83	
No	93	115	
Exercise			0.008
< 1 time/month	24	42	
< 1 time/week	66	50	
> 2 times/week	151	106	
Tea drinking habit			0.146
Yes	201	181	
No	30	17	

Note: OA, osteoarthritis.

### Structure changes of the normal articular cartilage tissues and OA articular cartilage tissues

**Figure 1:**
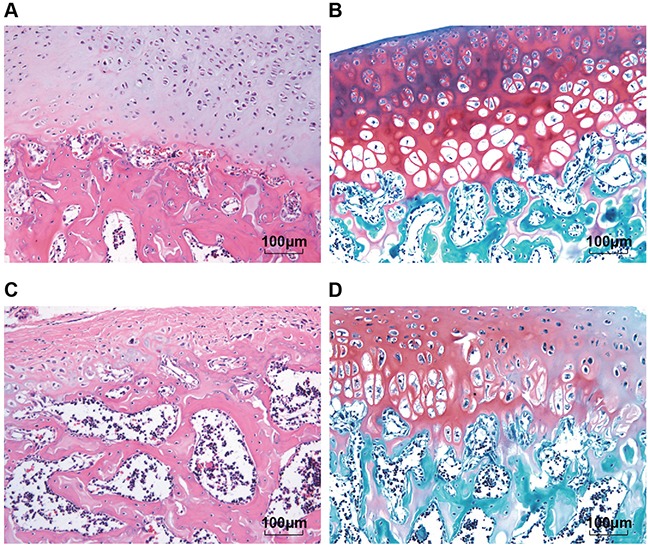
Comparisons of structure changes of the normal articular cartilage tissues and OA articular cartilage tissues according to the HE staining and safranin O/fast green FCF staining (200 ×) Notes: **(A)** and **(B)** were the result of normal cartilage tissues, respectively by HE staining and safranin O and fast green FCF staining; **(C)** and **(D)** were the result of OA cartilage tissues, respectively by HE staining and safranin O and fast green FCF staining; HE, Hematoxylin-Eosin; OA, osteoarthritis.

Structure changes of the normal articular cartilage tissues and OA articular cartilage tissues observed under the microscope were showed in Figure 1. According to the HE staining, the normal chondrocytes arranged in neat rows; number and morphology were normal; cartilage structure was clear and the tidal line was continuous and complete. While the OA chondrocytes showed hyperplasia, hypertrophy and disorder; they clustered or disappeared; the surface layer showed different degrees of damage, accompanied with angiogenesis. According to the results of safranin O/fast green FCF staining, with the aggravation of the lesion, the red area of the normal cartilage tissues turned light in color; the area of dyeing loss expanded until complete dyeing loss occurred; the tidal line was also damaged; new blood vessels were seen to connect marrow cavity through the tidal line. The cartilage pathological scoring results showed that there were 198 cases in the normal group, 104 cases and 127 cases in the OA mild lesion group and the OA moderate lesion group, respectively.

### The expressions of TLR-2, NF-κB and MMP-13 among three groups

**Figure 2:**
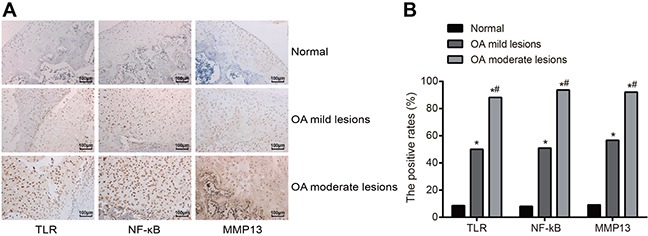
Comparisons of positive expressions of TLR-2, NF-κB and MMP-13 by immunochemistry among three groups Notes: **(A)** Detection of protein expressions of TLR-2, NF-κB and MMP-13 by immunochemistry among three groups (positive expression was presented as the brown yellow particles deposition, in the nucleus and part of cytoplasm of articular chondrocytes for TLR-2 and NF-κB and nucleus for MMP-13); **(B)** Positive protein expressions of TLR-2, NF-κB and MMP-13 in the chondrocytes by immunochemistry among three groups; *, compared with the normal group, *P* < 0.05; #, compared with the OA moderate lesion group, *P* < 0.05; TLR-2, toll-like receptor-2; NF-κB, nuclear transcription factor-kappa B; MMP-13, matrix metalloprotease-13; OA, osteoarthritis.

According to the results of the immunochemistry (Figure 2A), the positive expressions of TLR-2 and NF-κB were presented with brown yellow particles deposition in the nucleus of articular cartilage and a small amount in the cytoplasm of articular cartilage; while MMP-13 positive expression was presented with brown yellow particles deposition in the cytoplasm. Negative expression was indicated when only cell profile was observed, and the nucleus and cytoplasm were not colored. In the normal group, TLR-2, NF-κB and MMP-13 positively expressed in 17, 16 and 18 cases, independently, and the positive rates were 8.59%, 8.08% and 9.09%, respectively. In the OA mild lesion group, the cases of TLR-2, NF-κB and MMP-13 positively expressed were 52, 53 and 59, respectively, and the corresponding positive rates were 50.00%, 50.96% and 56.73%, respectively. In the OA moderate lesion group, the cases of TLR-2, NF-κB and MMP-13 positively expressed were 112, 119 and 117, respectively, and the corresponding positive rates were 88.19%, 93.70% and 92.13%, respectively. In pairwise comparisons, the difference of the positive expressions of TLR-2, NF-κB and MMP-13 was statistically significant (all *P* < 0.05); the positive expressions of TLR-2, NF-κB and MMP-13 were positively correlated with the degree of lesion (Figure 2B).

### Correlation between the expressions of TLR, NF-kB, MMP13 and clinicopathological characteristics of OA patients

Correlation between the expressions of TLR, NF-kB, MMP13 and clinicopathological characteristics of OA patients were shown in Table 2. The positive expressions of TLR, NF-kB, MMP13 were associated with BMI, family history, exercise, and WOMAC scores of OA patients (all *P* < 0.05), but age, gender, smoking, drinking and tea drinking habits showed no significant difference.

**Table 2:** Correlation between the expressions of TLR, NF-kB, MMP13 and clinicopathological characteristics of OA patients

Characteristic	TLR			NF-kB			MMP13		
	Positive	Negative	*P*	Positive	Negative	*P*	Positive	Negative	*P*
	Case	Case		Case	Case		Case	Case	
Age (years)			0.875			0.586			0.357
≥ 60	124	50		1248	46		130	44	
< 60	40	17		44	13		46	11	
Gender			0.071			0.489			0.117
Male	62	17		61	18		65	14	
Female	102	50		111	41		111	41	
BMI (kg/m^2^)			0.014			0.012			0.002
< 23	71	40		76	35		76	35	
23 ~ 25	45	19		46	18		48	16	
> 25	48	8		50	6		52	4	
Smoking			0.495			0.163			0.142
Yes	80	36		91	25		94	25	
No	84	31		81	34		82	34	
Drinking			0.584			0.891			0.199
Yes	55	25		60	20		57	23	
No	109	42		112	39		119	32	
Family history			0.038			< 0.001			0.002
Yes	105	33		117	21		115	23	
No	59	34		55	38		61	32	
Exercise			0.008			0.022			0.044
Few	11	13		18	6		19	5	
A few	45	21		41	25		43	23	
Often	108	33		113	28		114	27	
Tea drinking habit			0.575			0.88			0.948
Yes	20	10		22	8		23	7	
No	144	57		150	51		153	48	
WOMAC scores			0.01			0.027			0.023
Mild	72	46		79	39		81	37	
Moderate	73	20		76	17		78	15	
Severe	19	1		17	3		17	3	

Note: TLR-2, toll-like receptor-2; NF-κB, nuclear transcription factor kappa B; MMP-13, matrix metalloprotease-13; OA, osteoarthritis.

### Logistic regression analysis of the risk factors of OA

Whether having OA or not was served as the dependent variable and the age, sex, BMI, smoking, drinking, family history, exercise, and drinking habits were included in logistic regression analysis. And the results showed that TLR, NF-kB, MMP13, BMI, family history and exercise were related to OA (all *P* < 0.05), in which LR, NF-kB, MMP13, BMI and family history were risk factors (all EXP(B) > 1, *P* < 0.05) and exercise was protection factor (EXP(B) < 1, *P* < 0.05). Also, the age, sex, smoking, drinking, drinking habits and OA had no obvious correlation (all *P* > 0.05) (Table 3).

**Table 3:** Logistic regression analysis of the factors related to OA

Factor	B	S.E.	Wald	Sig,	Exp (B)	95% C.I
Age (years)	0.24	0.27	0.78	0.376	1.27	0.75 - 2.15
Gender	−0.41	0.25	2.76	0.097	0.66	0.41 - 1.08
BMI (kg/m^2^)						
< 23			10	0.007		
23 ~ 25	0.6	0.31	3.8	0.051	1.83	1.00 - 3.35
> 25	1.17	0.37	10	0.002	3.23	1.56 - 6.70
Smoking	0.19	0.24	0.63	0.428	1.21	0.76 - 1.92
Drinking	0.42	0.26	2.62	0.106	1.52	0.92 - 2.53
Family history	0.65	0.27	5.92	0.015	1.92	1.14 - 3.26
Exercise						
< 1 time/month			12.17	0.002		
< 1 time/week	−1.07	0.37	8.34	0.004	0.34	0.16 - 0.71
> 2 times/week	0.28	0.28	0.99	0.321	1.32	0.76 - 2.27
Tea drinking habit	0.37	0.4	0.87	0.352	1.45	0.67 - 3.14
TLR	1.23	0.25	24.48	< 0.001	3.42	2.10 - 5.56
NFKB	0.92	0.26	12.51	< 0.001	2.52	1.51 - 4.20
MMP13	1.37	0.25	29.35	< 0.001	3.93	2.39 - 6.44

Note: TLR-2, toll-like receptor-2; NF-κB, nuclear transcription factor kappa B; MMP-13, matrix metalloprotease-13; OA, osteoarthritis; B: constant value; S.E.: standard error; Sig: significant; CI: confidence interval.

### The mRNA expressions of TLR-2, NF-κB and MMP-13, IL-1, IL-6 and TNF-α among three groups

**Figure 3:**
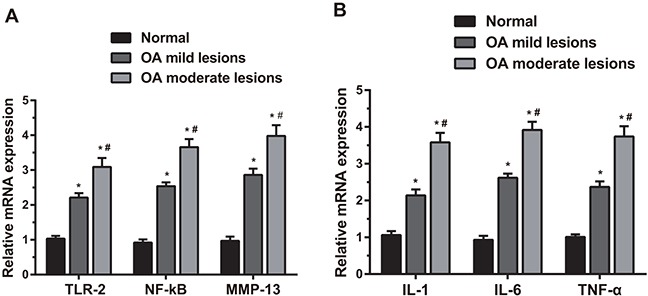
Comparisons of mRNA expressions of TLR-2, NF-κB, MMP-13, IL-1, IL-6 and TNF-α in the cartilage tissues by qRT-PCR among three groups Notes: (A). Comparisons of mRNA expressions of TLR-2, NF-κB and MMP-13 among three groups; (B). comparisons of mRNA expressions of IL-1, IL-6 and TNF-α among three groups; *, compared with the normal group, *P* < 0.05; #, compared with the OA moderate lesion group, *P* < 0.05; OA, osteoarthritis.

As shown in Figure 3A, compared with the normal group, the OA mild and moderate lesion groups showed significantly increased mRNA expressions of TLR-2, NF-κB and MMP-13 (all *P* < 0.05); the OA moderate lesion group showed significantly higher mRNA expressions of TLR-2, NF-κB and MMP-13 than those in the OA mild lesion group (all *P* < 0.05). The mRNA expressions of the related inflammatory factors are shown in Figure 3B, specifically, compared with the normal group, the OA mild and moderate lesion groups showed significantly increased mRNA expressions of IL-1, IL-6 and TNF-α (all *P* < 0.05); the OA moderate lesion group showed significantly higher mRNA expressions of IL-1, IL-6 and TNF-α than those in the OA mild lesion group (all *P* < 0.05). The mRNA expressions of TLR-2, NF-κB, MMP-13, IL-1, IL-6 and TNF-α were positively correlated with the degree of lesion.

### Isolation, culture and identification of chondrocytes

**Figure 4:**
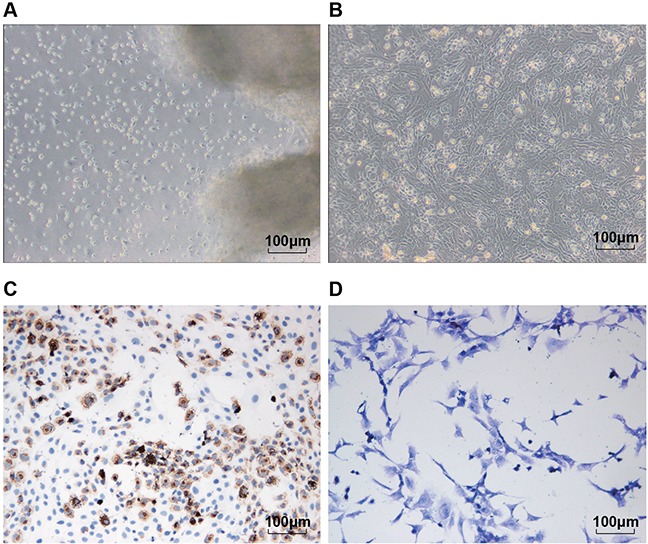
Isolation, culture and identification of chondrocytes Notes: **(A)** and **(B)** were respectively the primary and first generation of the separated chondrocytes (×100); **(C)** type II collagen staining results of the chondrocytes (×200); **(D)** toluidine blue staining results of the chondrocytes (×200).

Primary human chondrocytes were presented in a triangle with the cell cluster as the center, or in irregular shape; they crawled around. The chondrocytes of the first generation had less cluster compared with the primary cells, which with uniform morphology; the cells were slightly sparse in density, and with pseudopodia connected cells (Figure 4A, 4B). Type 2 collagen usually was presented in brown yellow after immunochemistry; we observed quantity of brown yellow particles in the cytoplasm and surrounding of chondrocytes (Figure 4C). According to the toluidine blue staining, the chondrocytes were mainly in light blue, which indicated the existence of proteoglycan and other extracellular matrix secreted by the chondrocytes (Figure 4D).

### Comparisons of the proliferation of chondrocytes among four groups

**Figure 5:**
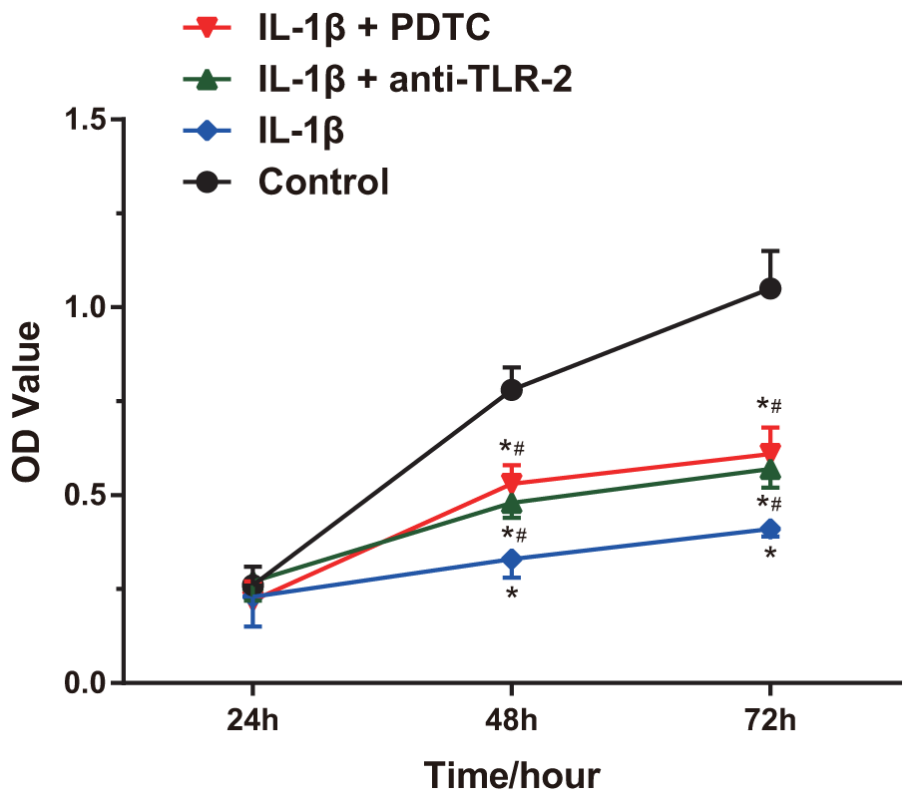
Comparisons of proliferation of chondrocytes among four groups Notes: IL-1β significantly inhibited cell growth; *, compared with the control group, *P* < 0.05; #, compared with the IL-1β group, *P* < 0.05; OD, optic density.

According to the MTT assay (Figure 5), the OD value which indicated proliferation of cells in each group was not statistically significant at 24 h (all *P* > 0.05). At 48 h and 72 h, the chondrocytes in the IL-1β group, the IL-1β + anti-TLR-2 group and the IL-1β + PDTC group were significantly inhibited in growth compared with the control group (all *P* < 0.05). At 48 h and 72 h, the chondrocytes in the IL-1β + anti-TLR-2 group and the IL-1β + PDTC group showed faster cell growth compared with those in the IL-1β group (all *P* < 0.05). The chondrocytes in the IL-1β + anti-TLR-2 group and the IL-1β + PDTC group showed no significant difference in proliferation at the three time points (all *P* > 0.05).

### Comparisons of cell apoptosis of chondrocytes among four groups

**Figure 6:**
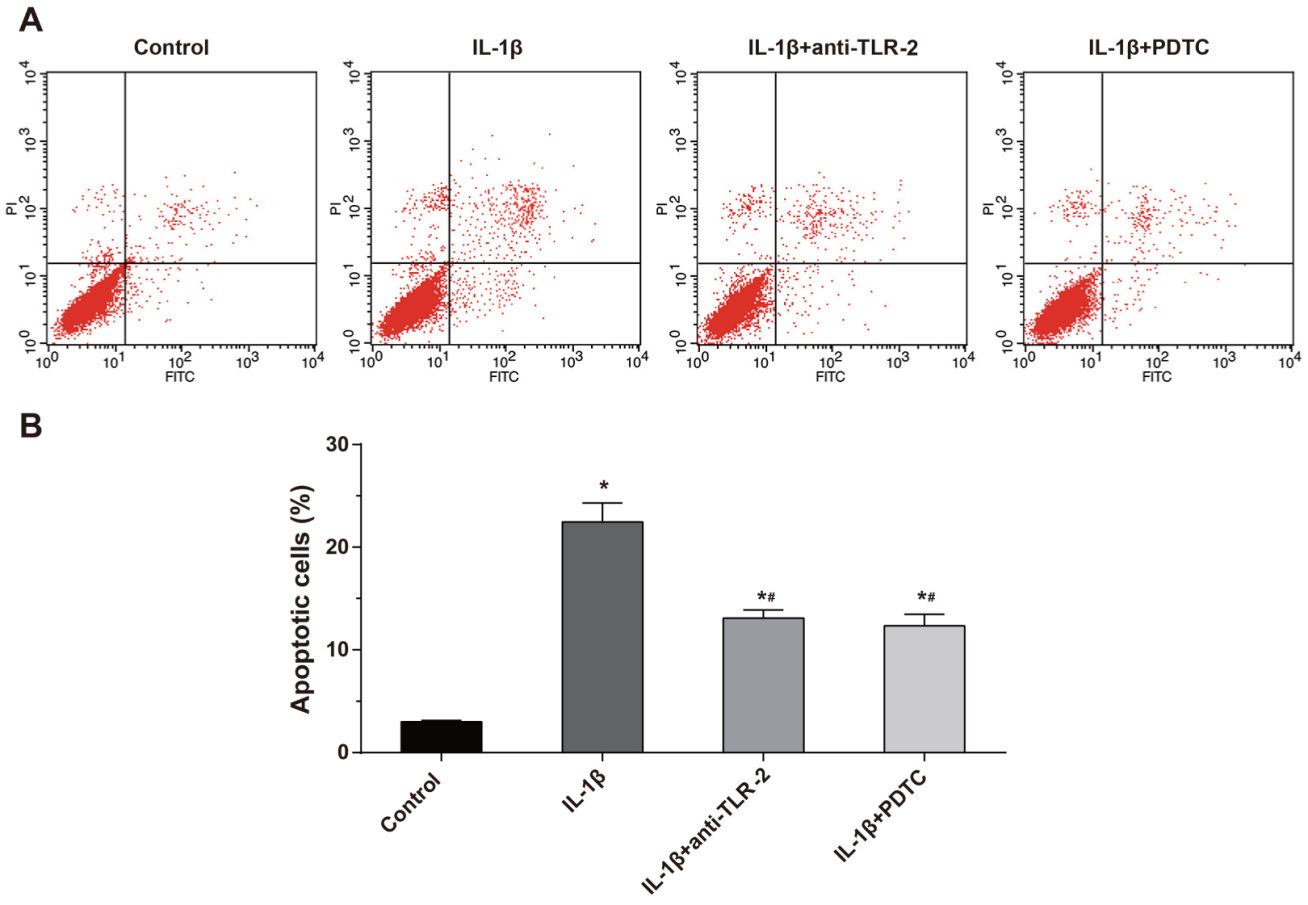
Comparisons of apoptosis of chondrocytes by Annexin V/PI method among four groups Notes: **(A)** Apoptosis of chondrocytes among four groups by flow cytometry; **(B)** histogram of the apoptosis rate in each group; *, compared with the control group, *P* < 0.05; #, compared with the IL-1β group, *P* < 0.05.

According to the Annexin V/PI method (Figure 6), the apoptosis rates of the cells in the control group, the IL-1β group, the IL-1β + anti-TLR-2 group and the IL-1β + PDTC group after treated 48 h were (2.97 ± 0.33)%, (22.15 ± 2.17)%, (13.72 ± 1.13)% and (12.54 ± 1.02)%, respectively. Whether interfered by anti-TLR-2 and PDTC or not, the apoptosis rate of the chondrocytes in the IL-1β group increased significantly compared with the control group (all *P* < 0.05). The apoptosis rate of the chondrocytes in the IL-1β + anti-TLR-2 and IL-1β + PDTC groups decreased significantly compared with the IL-1β group (all *P* < 0.05). The IL-1β + anti-TLR-2 group and the IL-1β + PDTC group showed no significant difference in the apoptosis rate of the chondrocytes.

### Comparisons of the protein expressions of TLR, NF-kB, p-NF-kB and MMP13 among four groups

**Figure 7:**
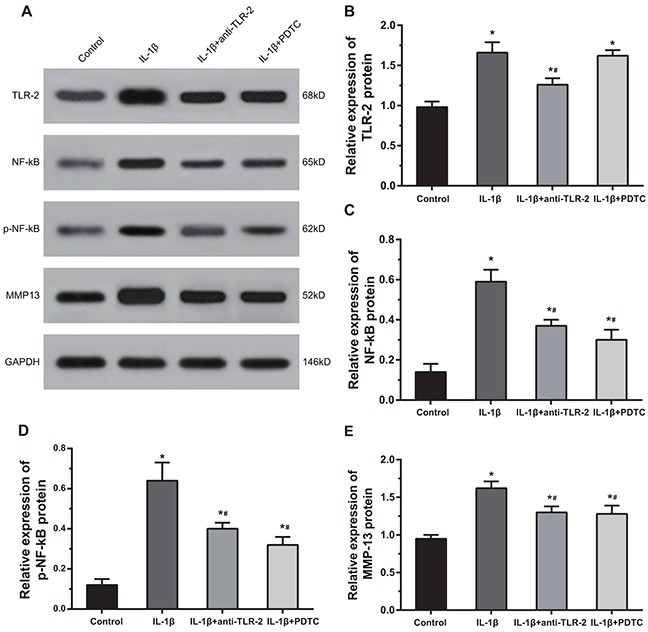
Comparisons of protein expressions of TLR-2, NF-κB, p-NF-κB and MMP-13 by Western blotting among four groups Notes: **(A)** Protein bands of TLR-2, NF-κB, p-NF-κB and MMP-13 in chondrocytes among four groups; **(B-E)** histograms of the protein expressions TLR-2, NF-κB, p-NF-κB and MMP-13 in chondrocytes; p-NF-κB, phosphorylated- nuclear transcription factor-kappa B; *, compared with the control group, *P* < 0.05; #, compared with the IL-1β group, *P* < 0.05.

As shown in Figure 7, compared with the control group, the IL-1β group, the IL-1β + anti-TLR-2 group and the IL-1β + PDTC group showed significantly increased protein expressions of TLR-2, NF-κB, p-NF-κB and MMP-13 (all *P* < 0.05). Compared with the chondrocytes in the IL-1β group, the cells in the IL-1β + anti-TLR-2 and IL-1β + PDTC groups showed decreased protein expressions of NF-κB, p-NF-κB and MMP-13 (all *P* < 0.05); the cells in the IL-1β + anti-TLR-2 group showed decreased protein expression of TLR-2 (*P* < 0.05); the cells in the IL-1β + PDTC group showed no significant difference in the protein expression of TLR-2. The IL-1β + anti-TLR-2 group and the IL-1β + PDTC group showed no significant difference in the protein expressions of TLR-2, NF-κB, p-NF-κB and MMP-13.

### Comparisons of the expressions of IL-1, IL-6 and TNF-α among four groups

**Figure 8:**
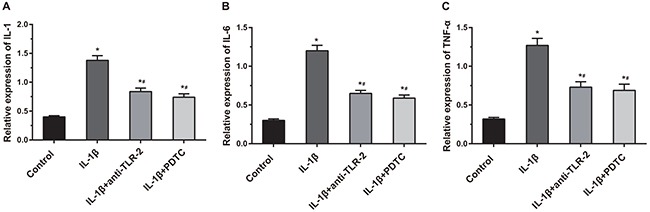
Comparisons of related inflammatory factors (IL-1, IL-6 and TNF-α) among four groups Notes: **(A)** Histogram of IL-1; **(B)** histogram of IL-6; **(C)** histogram of TNF-α; *, compared with the control group, *P* < 0.05; #, compared with the IL-1β group, *P* < 0.05.

As shown in Figure 8, compared with the control, IL-1β, IL-1β + anti-TLR-2 and IL-1β + PDTC groups showed significantly increased expressions of IL-1, IL-6 and TNF-α (all *P* < 0.05). Compared with the IL-1β group, the cell supernatants in the IL-1β + anti-TLR-2 and IL-1β + PDTC groups showed obvious decreased expressions of IL-1, IL-6 and TNF-α (all *P* < 0.05). The cell supernatant in the IL-1β + anti-TLR-2 and IL-1β + PDTC groups showed no significant differences in the expressions of IL-1, IL-6 and TNF-α.

## DISCUSSION

Progressive loss of articular cartilage in OA patients was recognized to be attributed to an interactional imbalance of anabolic, anti-catabolic, anti- and pro-inflammatory and anti- and pro-apoptotic activities [27–29]. By comparing the normal and OA cartilage tissues and cells, we explored the role of TLR-2/NF-κB signaling pathway in OA and the expressions of related inflammatory factors, with the main conclusion that expressions of TLR-2, NF-κB and MMP-13 and related inflammatory factors were up-regulated with the increase of degree of OA lesions, indicating that TLR-2/NF-κB signaling pathway can contribute to occurrence of OA.

Our study has illustrated that the positive expressions of TLR, NF-kB, MMP13 were associated with BMI, family history, exercise, and WOMAC scores of OA patients. As a degenerative joint disease, OA is characterized by progressive articular cartilage destruction, which leads to pain, joint swelling and reduced mobility [7]. Chondrocytes, stimulated by cytokines and growth factors, are the sole cells residing in articular cartilage, and maintain ECM through a homeostatic balance between anabolic and catabolic activities [30, 31]. Aberrances in the patterns of gene expression of chondrocytes, including expression increase of catabolic cytokines and degradative enzymes, which can cause destruction of the ECM [32]. Inflammation has been implicated in the pathogenesis of OA by shifting the balance from the anabolic toward catabolic state with progressive cartilage loss [33]. IL-1β, a pro-inflammatory cytokine, is recognized as a primary instigator in cartilage degradation in OA [34, 35]. Previous studies have illustrated that TLR2 expression increases in OA chondrocytes [24, 36]. IL-1β functions through autocrine and paracrine signaling pathways to promote synthesis of degradative enzymes, including MMPs and aggrecanases, which break down ECM actively [37]. MMP-13 can degrade aggrecan and the proteoglycan molecule, playing a dual role in matrix destruction [38]. Increased IL-1β expression is primary course for OA [39, 40] which may be a marker of inflammation in OA; therefore, it is not difficult to understand the increase of MMP-13 in OA, as well as related inflammatory factors, including IL-1, IL-6 and TNF-α. As involved in immune responses, TLR-2 and NF-κB have no reason not to be found up-regulated in OA, an inflammatory environment. And it is self-explanatory that with the increase of the degree of the lesions, the expressions of TLR-2, NF-κB and the related inflammatory factors also increased. TLR-2 was reported to be expressed by articular chondrocytes and the expressions were increased in OA lesions [41].

To further explore the effects of TLR-2 and NF-κB on OA, we introduced IL-1β inducement. Observed from our results, the chondrocytes in the IL-1β group, IL-1β + anti-TLR-2 group and IL-1β + PDTC group were significantly inhibited in growth compared with the control group; while the chondrocytes in the IL-1β + anti-TLR-2 and IL-1β + PDTC groups showed faster cell growth compared with those in the IL-1β group. In addition, the apoptosis rate of the chondrocytes in the IL-1β + anti-TLR-2 and IL-1β + PDTC groups decreased significantly compared with the IL-1β group. Furthermore, the cell supernatant in the IL-1β + anti-TLR-2 and IL-1β + PDTC groups showed decreased expressions of IL-1, IL-6 and TNF-α, compared with that in the IL-1β group. IL-1β has been demonstrated to inhibit matrix protein synthesis and induce matrix degrading enzymes and other pro-inflammatory cytokines, including IL-6 [42, 43]. A previous study showed that mice with IL-1β or IL-6 knockout suffered from accelerated OA development, indicating their critical role in cartilage biology [33]. Ortved KF et al. used posttranscriptional silencing of IL-1β to control the catabolic cascade involved in OA and the activation of the catabolic cascade is crucial for degradation of cartilage [32]. Therefore, we used IL-1β inducement to help compare and observe the effects of TLR-2 and NF-κB on OA.

OA is characterized by degeneration of the articular cartilage [6]. The findings in our study suggested that TLR-2 and NF-κB could contribute to suppressing the growth and promoting the apoptosis of chondrocytes, indicating that inhibiting TLR-2/NF-κB signaling pathway might improve chondrocytes viability and antagonize chondrocytes damage induced by IL-1β. IL-1β has been implicated in the degeneration of articular cartilage for its induction of proteoglycan loss and matrix degradation [44]. It was reported that acute injury induced production of pro-inflammatory cytokines and catabolic enzymes, promoting chondrocyte apoptosis and degraded cartilage [45]. Pro-inflammatory cytokines, like IL-1β and TNF-α, were demonstrated to differentially regulate the apoptotic pathway in human chondrocytes [46]. TLR ligands could activate the synoviocytes, which could secrete pro-inflammatory cytokines and chemokines, such as TNF-α, IL-15, RANTES (regulated on activation normal T-cell expressed and secreted) and granulocyte and monocyte chemotactic protein 2, contributing to synovitis maintenance and inflammatory cell infiltration [47–49]. Activated synoviocytes could also produce MMPs and RANKL (receptor activator of NF-κB ligand), which are involved in cartilage degradation and joint destruction [50–52]. Therefore, TLRs may be critical in mediating synovial inflammation in experimental arthritis [53]. Zhu W et al. demonstrated that the induction of TLR-3 in fibroblast-like synoviocytes (FLSs) was due to T cell-derived inflammatory stimulation and further mediated FLS activation in arthritis [47].

Stimulation of TLRs with their ligands could activate NF-κB [54]. NF-κB activation is known to be pro-inflammatory [17]. And the suppression of NF-κB has been related with anti-inflammatory activity [55]. Accumulating studies reported that TLR-mediated responses were key inducers of pro-inflammatory cytokines, which directly or indirectly resulted in development of lymphatic pathology [56–59]. Studies reported that ligands for TLRs (including TLR-2, 3, 4, 9) induced or exacerbated arthritis in experimental models[52, 60]. NF-κB has been reported to regulate expressions of genes involved in OA [61]. TLR-2/NF-κB signaling pathway has been implicated in many studies related with inflammation or immune [62–64]. The specific mechanism behind how it affected the proliferation and apoptosis of chondrocytes was not elucidated yet and needed further exploration.

In conclusion, the expressions of TLR-2, NF-κB and MMP-13 and related inflammatory factors increased with the increase of degree of OA lesions, indicating that TLR-2/NF-κB signaling pathway can contribute to occurrence of OA; inhibiting TLR-2/NF-κB signaling pathway. However, the inner mechanisms of related inflammatory factors and degenerative OA are still unclear, more researches are needed to be conducted to provide a target for the degenerative OA treatment.

## MATERIALS AND METHODS

### Ethic statement

The study was approved by the Ethics Committee of Huaihe Hospital of Henan University and all the study subjects have signed an informed consent.

### Sample collection

A total of 231 patients with degenerative OA receiving total knee arthroplasty (TKA) in Huaihe Hospital of Henan University from January 2013 to December 2015 were selected for our study. The diagnosis was in accordance with the diagnostic criteria for knee OA in 2001 revised by American College of Rheumatology (ACR; formerly, the American Rheumatism Association) [65]. Among these 231 patients, there were 79 males and 152 females, with age ranging from 36 to 96 years old and the mean age 65.42 ± 8.74 years old. Clinical criteria: (1) Most of time nearly 1 with knee pain; (2) With bony crepitus; (3) early morning stiffness ≤ 30 min; (4) Age ≥ 38; (5) With bony enlargement. Clinical and radiological criteria: (1) Most of time nearly 1 with knee pain; (2) X-ray forming osteophyte; (3) Joint fluid examination consistent with OA; (4) Age ≥ 40; (5) early morning stiffness ≤ 30 min; (6) With bony crepitus. Knee OA can be diagnosed when conforming to 1 + 2 or 1 + 3 + 5 + 6 or 1 + 4 + 5 + 6. The articular cartilage samples were collected from patients with traumatic amputation as OA group. Totally 198 patients received amputation because of trauma in the Emergency Department of our hospital, including 76 males and 122 females, with age ranging from 38 to 81 years old and the mean age 64.03 ± 7.70 years old. The articular cartilages were collected for sampling as the normal group. Patients with obvious osteoporosis or neoplastic lesion or non-primary arthritis, for example, rheumatoid arthritis, suppurative arthritis or trauma-induced arthritis, were excluded.

### Histological staining

Cartilage block with subchondral bone was cut into 1.0 cm × 1.0 cm × 0.5 cm sized blocks, which were then fixed for 3 days with neutral formalin solution, followed by decalcification for 14 days in 30% formic acid solution, dehydration with ethanol in conventionally gradient, paraffin embedding, cutting into 5 μm slices and drying for further use. The Hematoxylin-Eosin (HE) staining was performed as follows. After dewaxing and hydration, the cartilage samples were stained for 5 min in Harris alum hematoxylin (Fuzhou Maixin Biotechnology, Co., Ltd., Fuzhou, China), followed by washing and color separation for 10 s in 0.5% hydrochloric acid alcohol. After washing, the samples were stained for 40 s in eosin (Fuzhou Maixin Biotechnology, Co., Ltd., Fuzhou, China), followed by dehydration, transparency, mounting with neutral balsam. Observed under a microscope, the nucleus of the chondrocytes appeared blue and the other tissues appeared pink. The procedures of safranin O/fast green FCF staining: After dewaxed and hydrated, the cartilage samples were stained for 5 min in Harris alum hematoxylin, followed by washing and color separation for 10 s in 0.5% hydrochloric acid alcohol. After washing again, the samples were placed in 0.2% fast green solution (Shanghai Sangon Biological Engineering Technology & Services Co., Ltd., Shanghai, China) for 1 min, 1% ethylic acid solution for 30 s and 0.1% safranin O solution (Shanghai Sangon Biological Engineering Technology & Services Co., Ltd.) for 15 min, followed by dehydration, transparency and mounting with neutral balsam. The normal cartilage appeared red and the background appeared green. According to the improved Mankin cartilage pathological score standard [66], the articular cartilage samples were graded from four aspects, namely cartilage structure, cartilage cell morphology, the results of safranin O/fast green FCF staining and tidal line, as the normal group (0 ~ 2 points), the OA mild lesion group (3 ~ 7 points) and the OA moderate lesion group (8 ~ 11 points).

### Immunohistochemistry

After fixation and decalcification, the samples were cut into conventional slices, followed by dewaxing with xylene and gradient alcohol hydration. The slices were added with drops of 3% H_2_O_2_ and then rested for 15 min to block endogenous peroxidase, followed by washing with phosphate buffered saline (PBS) for three times. The cartilage slices were added with heated sodium citrate buffer (10 mM) and microwaved for 6 min for antigen repair. The procedure was repeated twice, followed by cooling and washing with PBS. The cartilage slices were then treated with 5% goat serum for 10 min, in order to obstruct the binding of nonspecific antibody. After added with drops of the first antibodies of TLR-2, NF-κB and matrix metalloprotease-13 (MMP-13) (Cell Signaling Technologies, Beverly, MA, USA), the slices were incubated at 37°C for 30 min and washed with PBS. After being added with horse radish peroxidase labelled second antibody (Cell Signaling Technologies, Beverly, MA, USA), the slices were incubated at 37°C for 30 min and washed with PBS, followed by staining for 10 min with diaminobenzidine (DAB) (Sigma-Aldrich Chemical Company, St Louis MO, USA), washing with running water, re-staining with hematoxylin, mounting with neutral balsam and lastly observing under a microscope and picture-taking.

### Quantitative real-time polymerase chain reaction (qRT-PCR)

The tissue samples were cut into thin slices and then grinded using a liquid nitrogen method. According to the reagent specification of Trizol (Invitrogen Inc., Carlsbad, CA, USA), the total RNA of the tissue samples were extracted in Trizol one-step method. Reverse transcription of the RNA was performed in a two-step method following the reagent specification (Fermentas Inc., Hanover, MD, USA). The reaction conditions were as follows: 70°C for 10 min, ice-bath for 2 min, 42°C for 60 min and 70°C for 10 min. The cDNA obtained was reserved temporarily in a refrigerator at −80°C. The qRT-PCR was carried out using TaqMan probing method. The reaction system was operated according to the reagent specification (Fermentas Inc., Hanover, MD, USA). The primer sequences are shown in Table 4. The reaction conditions: pre-denaturation at 95°C for 30 s, denaturation at 95°C for 10 s, annealing at 60°C for 20 s and extension at 70°C for 10 s, with totally 40 cycles. qRT-PCR instrument (Bio-Rad, Inc., Hercules, CA, USA; model: Bio-Rad iQ5) was applied for detection, with β-actin as an internal reference for target gene, relative quantitative method for calculating and 2-ΔΔCt representing relative expression multiple of each target gene. Each experiment was repeated 5 times.

**Table 4:** Primer sequences of qRT-PCR

Targetgene	Primer sequence
TLR-2	F: 5′-CCTGTGCAATTTGACCATTG-3′
	R: 5′-AAGCATTCCCACCTTTGTTG-3′
NF-κB	F: 5′-CCTGGATGACTCTTGGGAAA-3′
	R: 5′-TCAGCCAGCTGTTTCATGTC-3′
MMP-13	F: 5′-TGAGAGTCATGCCAACAAATTC-3′
	R: 5′-CAGCCACGCATAGTCATGTAGA-3′
IL-1	F: 5′-GGACAAGCTGAGGAAGATGC-3′
	R: 5′-TCCATATCCTGTCCCTGGAG-3′
IL-6	F: 5′-GAGCTTCAGGCAGGCAGTATC-3′
	R: 5′-GTATAGATTCTTTCCTTTGAGGC-3′
TNF-α	F: 5′-TCAGAGGGCCTGTACCTCAT-3′
	R: 5′-GGAAGACCCCTCCCAGATAG-3′
β-actin	F: 5′-AGCGAGCATCCCCCAAAGYY-3′
	R: 5′-GGGCACGAAGGCTCATCATT-3′

Note: qRT-PCR, quantitative real-time polymerase chain reaction; TLR-2, toll-like receptor-2; NF-κB, nuclear transcription factor kappa B; MMP-13, matrix metalloprotease-13; IL-1, interleukin-1; IL-6, interleukin-6; TNF-α, tumor necrosis factor alpha; F, forward; R, reverse.

### Isolation, culture and detection of chondrocytes

The cartilage collected from the OA patients in the surgery was cut into sections sized 1 ~ 2 mm^3^, followed by PBS washing and 0.25% type II collagen enzyme (Sigma-Aldrich Chemical Company) digestion overnight. The digestive juice was filtered by a 200 mesh cell filter (Corning Glass Works, Corning, N.Y., USA), and then the chondrocytes were centrifuged at 1000g for 5 min. After discarding the supernatant, the cells were added with Dulbecco minimum essential medium (DMEM) containing 10% fetal bovine serum (FBS) (Gibco Company, Grand Island, NY, USA), followed by suspension and culture at 37°C with 5% CO_2_. After growing full of the incubator, the chondrocytes were digested with 0.25% pancreatic enzyme (Gibco Company) and cultured for passage. Well-grown chondrocytes were collected to make climbing films, which were stained with toluidine blue (Beijing Solarbio Science & Technology Co., Ltd., Beijing, China) according to the reagent specification of immunochemistry staining, and labelled with type II collagen (USCN Life Science & Technology Company, Double Lake, Missouri City, TX, USA), followed by dehydration with absolute alcohol, mounting with neutral balsam and observing under a microscope and picture-taking.

### Cell grouping

The isolated and cultured chondrocytes were inoculated into corresponding plates or culture dishes at an appropriate density at 37°C with 5% CO_2_. After adherent, the cells were treated for 48 h with antibody of interleukin-1β (IL-1β; at 10 ng/ml; Sigma-Aldrich Chemical Company) and TLR-2 (at 50 ng/ml; Sigma-Aldrich Chemical Company), and NF-κB inhibitor pyrrolidinedithiocarbamic acid (PDTC; 100 uM; Sigma-Aldrich Chemical Company). The cells were divided into 4 groups, namely the control group, the IL-1β group, the IL-1β + anti-TLR-2 group and the IL-1β + PDTC group.

### MTT assay

The chondrocytes were inoculated into a 96-well plate (10^4^ cells in each well). The three experiment groups were treated respectively with anti-TLR-2, NF-κB inhibitor PDTC and IL-1β; and three wells were set for each group. After being incubated for a period of time at 37°C with 5% CO_2_, each well was added with 20 μL MTT solution (5 mg/ml; Sigma-Aldrich Chemical Company) and cultured for another 4 h. After the supernatant was discarded, each well was added with 150 μL of dimethylsulfoxide (DMSO), followed by low-speed shaking for 10 min. Enzyme-linked immunosorbent assay (ELISA) was performed to detect the optical density (OD) value at the wavelength of 490 nm.

### Flow cytometry

After treated for 48 h, the cells were collected into flow tubes and then centrifuged for 5 min at 1000r/min, and then the supernatant was discarded. The cells were washed with cooled PBS for three times and centrifuged, and then the supernatant was discarded. According to the specification of Annexin-V-FITC cell apoptosis detection Kit (Sigma-Aldrich Chemical Company), each tube was added with 150 μL of binding buffer and 5 μL of Annexin-V-FITC, followed by shaking and mixing, and incubation for 15 min in dark at room temperature. The addition of 100 μL of binding buffer and 5 μL of propidium iodide (PI; Sigma-Aldrich Chemical Company) and evenly mixing were conducted. A flow cytometry was used to determine cell apoptosis.

### Western blotting

The chondrocytes treated for 48 h were collected and washed for three times with pre-cooling PBS, then lysed with protein extraction lysis solution (Beyotime Biotechnology Co., Shanghai, China) and lastly rested on ice for 30 min, followed by centrifugation at 12000g for 10 min at 4°C, separate packing and reservation at −20°C. Bovine Serum Albumin (BSA) protein with gradient density (Beijing Solarbio Science & Technology Co., Ltd., Beijing, China) was prepared; the protein concentration of each sample was determined according to Pierce BCA Protein Assay Kit (Thermo Fisher Scientific Inc., Waltham, MA). Electrophoresis was performed firstly at 60 V and then 120 V after the protein samples entered into separation gel, for 1 ~ 2 h, at 4°C in a cold room. After electrophoresis, the protein samples were transferred to polyvinylidene fluoride (PVDF) membrane using wet transferring method, for 2 h, at 4°C in a cold room. After removing the PVDF membrane, the protein samples were sealed with 5% evaporated milk and Tris-buffered saline-Tween 20 (TBST), followed by incubation for 1 ~ 2 h at room temperature. After added with the first antibodies of TLR-2, NF-κB, phosphorylated-NF-κB (p-NF-κB) and MMP-13 (Cell Signaling Technologies), the protein samples were rested overnight at 4°C, followed by washing with TBST (3 × 10 min), addition of mouse second antibody (Abcam Inc., Cambridge, MA, USA), resting for 1 h at room temperature and washing with TBST (3 × 10 min). Chemiluminescence, X-ray film tableting, developing and fixing were carried out. All the bands were analyzed for relative OD value, with the ratio of the integrated OD value of the target band and the integrated OD value of the corresponding internal reference GAPDH as the relative expression of the target protein.

### ELISA

ELISA was performed to determine the cell inflammation related factors, including IL-1, IL-6 and TNF-α. The chondrocytes at the logarithmic growth phase were inoculated into a 96-well plate. After the experiment groups were treated for 48 h, the cell supernatant was collected for detection. After added with diluted 100 μL of standard or sample to be tested and completely mixed, the cell supernatant was cultured for 120 min at 37°C. The reaction plate was fully washed and then dried. Each well was then added with 100 μL of enzyme conjugate (IL-1, IL-6 and TNF-α) (Beijing QiWei YiCheng Tech Co., Ltd.), mixed and then cultured in dark for 10 min at 37°C. Each well was added with 100 μL of stop buffer. A microplate reader was applied to detect the OD value at the wavelength of 450 nm; the concentrations of IL-1, IL-6 and TNF-α were determined according to the standard curve.

### Statistical analysis

All the data were analyzed using SPSS 21.0 statistic software (IBM-SPSS Inc., Chicago, IL, USA). Measurement data were presented as mean ± standard deviation; comparison among groups was analyzed using one-step variance; comparison between two groups was testified using LSD-t. Enumeration data were presented as frequency. Comparison of categorical data was validated by chi-square and hierarchical data by rank-sum test. Logistic regression analyzed factors related to OA. *P* < 0.05 was considered significantly different.
